# The clinical value of long - term electroencephalogram (EEG) in seizure - free populations: implications from a cross-sectional study

**DOI:** 10.1186/s12883-019-1521-1

**Published:** 2020-03-12

**Authors:** Tang Xinghua, Li Lin, Fan Qinyi, Wei Yarong, Pu Zheng, Liu Zhenguo

**Affiliations:** Department of Neurology, Xinhua Hospital, Shanghai Jiao Tong University School of Medicine, Shanghai, China

**Keywords:** Epilepsy, LTM EEG, Seizure-free, Epileptiform activity

## Abstract

**Backgroud:**

This study aimed to explore the clinical value of long - term electroencephalogram (LTM EEG) in seizure-free individuals taking antiepileptic drugs (AEDs) for more than 2 years. We try to look for clinical factors associated with epileptiform activity on LTM EEG in seizure free patients. We hope that the detection of epileptiform activity by the LTM EEG recording can develop the better treatment strategy.

**Methods:**

The LTM EEG recordings of 770 individuals with a definite diagnosis of epilepsy were assessed. Two hundred sixty-two individuals accorded with the inclusion criteria and exclusion criteria. We collect the demographic and clinical information and LTM EEG data of these 262 individuals. We analysed the data by one-way analysis of variance and Cox proportional hazards models.

**Results:**

We found that more epileptiform activity were found with LTM EEG recording than regular EEG recording in seizure-free individuals. We found several clinical factors could be associated with epileptiform activity on LTM EEG in seizure free patients by a one-way analysis: symptomatic or cryptogenic epilepsy [hazard ratio (HR) = 2.6], history of cerebral trauma (HR = 7.5), and abnormal imaging findings (HR = 3.1). The following factors suggested a correlation between history of cerebral trauma (HR = 2.4) and history of cerebral surgery (HR = 3.4) with epileptiform activity on LTM EEG presentation by multivariate logistic regression analysis.

**Conclusions:**

The study indicated a correlation of a number of factors with abnormal LTM EEG presentation: symptomatic or cryptogenic epilepsy, history of cerebral trauma, history of cerebral surgery, and abnormal imaging findings. The LTM EEG recording may help find epileptiform activity in high risk seizure-free individuals. The individuals need be reevaluated the therapeutic strateagies, and increase the hope to reach real seizure-free.

## Introduction

Epilepsy is a chronic disease need long-term management. The probability of remaining seizure-free after treatment discontinuation is about 70% at 2 years [1]. There are still about 30% patients couldn’t get seizure-free, and approximately 20–57% individuals would have a seizure relapse in seizure-free patients [2–4]. Quite a lot of individuals with epilepsy recurrence were due to factors such as taking multiple AEDs, hippocampal atrophy [5], focal epilepsy, multiple types of seizures, focal epileptiform abnormalities on electroencephalography(EEG) presentation, and especially increased EEG abnormalities during or after AED discontinuation [2, 6]. Of these factors, only epileptiform activity could be monitored by long - term electroencephalogram (LTM EEG), which is sensitive to detect epileptiform activity. Therefore, it is necessary to follow up LTM EEG recording during long-term epilepsy management process and then adopt an appropriate drug withdrawal scheme for patients.

The LTM EEG recording includes ambulatory EEG (AEEG) and video LTM EEG (VEEG). They have a higher sensitivity and lower false-negative rate compared with regular EEG recording. They are more sensitive to detect epileptiform activity than the regular EEG [7, 8]. AEEG also has the benefit of home recording for monitoring, and VEEG can help doctors distinguish nocturnal seizure or artifact [9]. The LTM EEG help to detect sub-clinical episode, improve the diagnosis rate of epilepsy and obtain the real world evidence. Therefore, it is necessary to follow up LTM EEG recording during long-term epilepsy management process and then adopt an appropriate drug withdrawal scheme for patients. The role of LTM EEG recording in determining AED treatment or predicting the relapse risk has been evaluated in multiple studies [10, 11]. However, there have few researches been done on the risk factors of LTM EEG recording in seizure-free patients. Only one study showed female, delayed therapy, longer duration of intractability may have the relationship with abnormal ambulatory EEG presentation [12].

This cross-sectional study aimed to explore the clinical value of LTM EEG presentation in seizure-free individuals taking antiepileptic drugs (AEDs) for more than 2 years. Thus we hope that the detection of sub-clinical episode by the LTM EEG recording can develop the better treatment strategy and help patients obtain real seizure-free.

## Patients and methods

All patients were recruited from the Department of Neurology at Xinhua Hospital affiliated to Shanghai Jiao Tong University School of Medicine, Shanghai, China. Follow-up assessments of 770 epilepsy inpatients from the epilepsy center database were conducted from January 1, 2010, to December 31, 2016. Of these, 262 individuals accorded with the inclusion criteria and exclusion criteria. We followed up these patients in the inpatient ward or outpatient department, and we followed up them at least 2 yrs. All the individuals enrolled were seizure-free, and they did LTM EEG recording per 6-12 months. The data were collected from January 1, 2017, to June 1, 2017. These assessments were conducted by the epilepsy physician.

### Inclusion criteria

The inclusion criteria were as follows: (1) between 8 and 80 years of age; (2) a history of seizures according to the 1981 classification system of the International League Against Epilepsy (ILAE 1981, 3) diagnostic criteria for epilepsy:①At least two unprovoked (or reflex) seizures occurring greater than 24 h apart; ②One unprovoked (or reflex) seizure and a probability of further seizures similar to the general recurrence risk (at least 60%) after two unprovoked seizures, occurring over the next 10 years; (4) had received continuous treatment with a stable dose of one or more different AEDs; and (5) reported to be taking AEDs for at least 2 years and being seizure-free for at least 2 years.

### Exclusion criteria

The exclusion criteria were as follows: (1) younger than 8 or older than 80 years of age; (2) a history of irregular AED treatment; (3) had stopped taking AEDs; (4) lost contact during follow-up; (5) unable to complete LTM EEG recording; and (6) medical records missing.

### Demographic and clinical information

Data on age at onset of seizures, age, gender, course of disease, seizure-type classification, history of febrile convulsions, history of postpartum anoxic, history of cerebral trauma, history of cerebral surgery, family history of epilepsy, abnormal LTM EEG presentation, brain abnormalities revealed by neuroradiological assessment (computed tomography/magnetic resonance imaging), number and type of AEDs taken, and seizure-free period were collected.

**Fig. 1:**
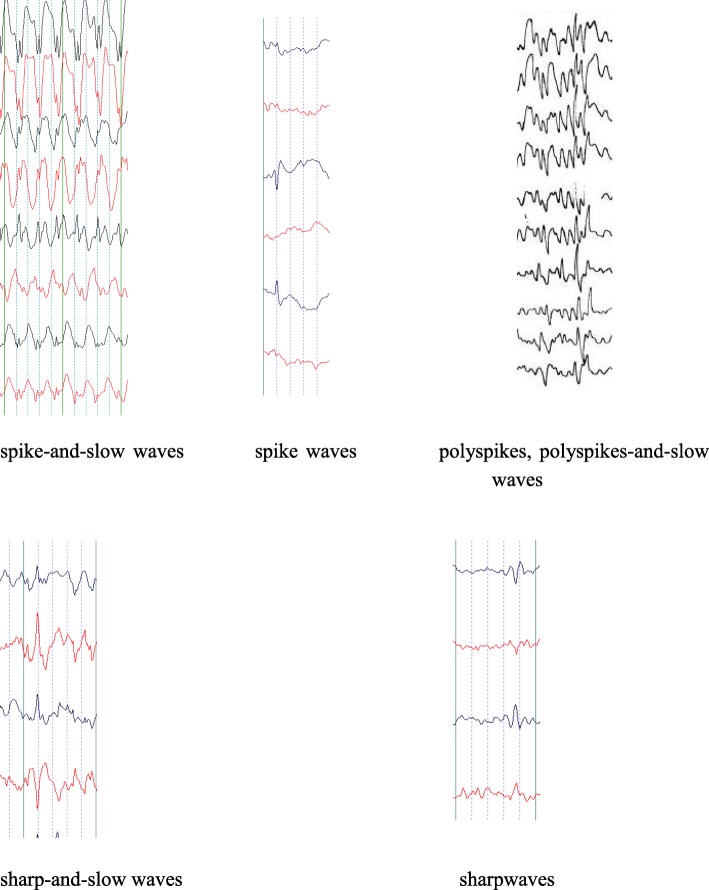
Abnormal LTM presentation (ictal activity)

An abnormal LTM EEG presentation was considered to ictal activity, include spikes, sharp waves, polyspikes, spike-and-slow waves, sharp-and-slow waves and polyspikes-and-slow waves without clinical seizures in this study (Fig. 1).

Brain abnormalities included brain tumors, cortical dysplasia, tuberous sclerosis, encephalitis, brain injuries, cerebral infarction, and cerebral hemorrhage.

### Statistical analysis

The data were processed using the SPSS statistical package (SPSS, IL, USA). The demographic and clinical characteristics of the patients were evaluated using the Student *t* test for continuous variables and the *χ*^*2*^ test for categorical variables. Logistic regression and Cox proportional hazards models were used to identify clinical factors associated with epileptiform activity on LTM EEG presentation. Hazard ratios (HRs) and 95% confidence intervals (CIs) were derived from Cox proportional hazard models. Logistic regression and Cox models met the assumption of proportionality of risks.

## Results

### Demographic and clinical characteristics

A total of 770 individuals were enrolled, and their AEEG/VEEG was followed up between January 1, 2010, and December 31, 2016. Of these, 357 individuals (46.4%) had been seizure-free for more than 2 years. Of these seizure-free patients, 262 individuals (73.4%) (121 females and 141 males, Table 1) still took AEDs, including 68 with VEEG recording and 194 with AEEG recording. Further, 262 individuals had done more than one LTM EEG recording, and 75 still had abnormal EEG presentation. The mean seizure-free duration in these individuals was 49.0 ± 29.0 months; it was 50.7 ± 31.5 months in individuals with abnormal EEG presentation and 48.4 ± 28.0 months in individuals with normal EEG presentation. The average age at onset of all 262 individuals was 13.7 ± 12.7 years; it was 12.4 ± 11.7 years in individuals with abnormal EEG presentation and 14.3 ± 13.1 years in individuals with normal EEG presentation. The duration of disease in all 262 individuals was 84.6 ± 55.9 months; it was 90.3 ± 74.8 months in individuals with abnormal EEG presentation and 82.3 ± 46.3 months in individuals with normal EEG presentation. Of these, 83 individuals had taken one kind of AED, whereas 179 had taken more than one kind of AED. The general characteristics and clinical features of the individuals are shown in Table 1.

**Table 1:** Demographic and clinical characteristics in patients seizure-free ≧2 years

Variable	All (*n* = 262)	a = abnormal EEG presentation (*n* = 75)	b = normal EEG presentation (*n* = 187)
Gender(male: female)	141:121	40:35	101:86
Age$\left(\overline{x}\pm \mathrm{s},\mathrm{year}\right)$	21.7 ± 13.1	20.2 ± 12.9	22.3 ± 13.1
Age at onset$\left(\overline{x}\pm \mathrm{s},\mathrm{year}\right)$	13.7 ± 12.7	12.4 ± 11.7	14.3 ± 13.1
Course of disease (month)	84.6 ± 55.9	90.3 ± 74.8	82.3 ± 46.3
Seizure type classification			
Idiopathic epilepsy(%)	191 (72.9)	43 (57.3)	148 (79.1)
Symptomatic/ Cryptogenic epilepsy(%)	71 (27.1)	32 (42.7)	39 (20.9)
History of febrile convulsions(%)	32 (12.3)	10 (13.5)	22 (11.8)
History of postpartum anoxic	8 (3.1)	3 (4.1)	5 (2.7)
History of cerebral trauma	13 (5.0)	10 (13.5)	3 (1.6)
History of cerebral surgery	6 (2.3)	6 (8.1)	0
Family history of epilepsy(%)	8 (1.6)	5 (6.8)	3 (3.1)
Imaging findings(%)	29 (11.2)	18 (25.0)	11 (5.9)
Number of AEDs≧1(%)	43 (16.4)	16 (21.3)	27 (14.4)
Seizure-free period $\left(\overline{x}\pm \mathrm{s},\mathrm{month}\right)$	49.0 ± 29.0	50.7 ± 31.5	48.4 ± 28.0

### One-way analysis of variance

The following factors could contribute to the abnormal EEG presentation by one-way analysis of variance (ANOVA): (1) symptomatic epilepsy; (2) patients with a history of cerebral trauma; (3) patients with a history of cerebral surgery; (4) patients with a family history of epilepsy; and (5) patients with abnormal imaging findings (*P* = 0.001, 0.0001, 0.0001, 0.044, and 0.0001 respectively) (Table 2).

**Table 2:** Risk of abnormal EEG findings in patients seizure-free ≧2 years: one way ANOVA analysis

Variable	All (*n* = 262)	a = abnormal EEG presentation (*n* = 75)	b = normal EEG presentation (*n* = 187)	*T*/*χ*^*2*^ value(a vs b)	*P* valu*e*
Seizure type classification					
Idiopathic epilepsy(%)	191 (72.9)	43 (57.3)	148 (79.1)	12.9	0.001 ^a^
Symptomatic/ Cryptogenic epilepsy(%)	71 (27.1)	32 (42.7)	39 (20.9)		
History of cerebral trauma	13 (5.0)	10 (13.5)	3 (1.6)	15.6	0.001 ^a^
History of cerebral surgery	6 (2.3)	6 (8.1)	0 (0)	15.4	0.001 ^a^
Family history of epilepsy(%)	8 (1.6)	5 (6.8)	3 (3.1)	4.7	0.044 ^a^
Imaging findings(%)	29 (11.2)	18 (25.0)	11 (5.9)	19.0	0.001 ^a^

^a^ Statistically significant

Other factors, such as history of febrile convulsions, history of postpartum anoxia, and number of AEDs taken by the patients did not show a statistically significant correlation with abnormal EEG presentation.

### Cox proportional hazard ratios

**Fig. 2:**
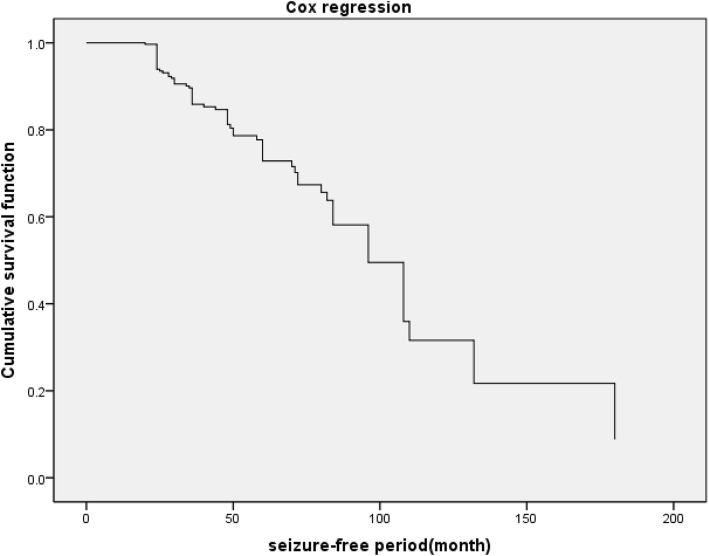
Risk of abnormal EEG seizure-free ≧2 years: Cox proportional hazard ratios

Two factors were found to have a correlation with LTM EEG presentation: (1) history of cerebral trauma (*p* = 0.019, HR = 2.42, 95% CI = 1.16–5.04, 2) history of cerebral surgery (*p* = 0.011, HR = 3.36, 95% CI = 1.31–8.6) (Table 3 and Fig. 2). Another two factors having a tendency to influence LTM EEG presentation were as follows: (1) abnormal imaging findings (*p* = 0.053, HR = 1.86, 95% CI = 0.99–3.5, 2) symptomatic/cryptogenic epilepsy (*p* = 0.08, HR = 1.61, 95% CI = 0.94–2.75) (Table 3).

**Table 3:** Risk of abnormal EEG seizure-free ≧2 years: Cox proportional hazard ratios

Variable	B	SE	Wald	Sig.	Exp(B)	95.0% CI
History of cerebral trauma	0.88	0.38	5.5	0.019 ^a^	2.4	1.2–5.0
History of cerebral surgery	1.2	0.48	6.4	0.011 ^a^	3.4	1.3–8.6
Symptomatic/ Cryptogenic epilepsy(%)	0.48	0.27	3.1	0.080 ^b^	1.6	0.94–2.8
Imaging findings(%)	0.62	0.32	3.8	0.053 ^b^	1.9	0.99–3.5
History of febrile convulsions(%)	0.20	0.38	0.28	0.60	1.2	0.58–2.6
History of postpartum anoxic	0.87	1.02	0.73	0.39	2.4	0.043–13.7

^a^ Statistically significant

^b^ Tendency for statistical significance

B:Regression coefficient

SE:Standard error

Sig:Significance

Exp(B):OR, Odds ratio

95.0% CI:95.0% confidence interval

## Discussion

The aim of following up long-term EEG is to detect ictal activities and then adjust AED treatment for reducing the risk of relapse in seizure-free individuals. In the present study, we found that more epileptiform activity were found with LTM EEG recording than regular EEG recording in seizure-free individuals. Individuals with symptomatic/ cryptogenic epilepsy, or history of cerebral trauma, or abnormal imaging findings tend to have higher risk of abnormal LTM EEG presentation.

Three hundred fifty-seven individuals (46.4%) reached a seizure-free status in ≧2 years. Further, 262 individuals still continuously taking AEDs were enrolled. The remaining 95 seizure-free individuals did not take any AEDs. Although in these 262 individuals had a seizure-free status, 75 individuals (28.6%) had abnormal LTM EEG presentation. Several studies reported that 10–20% of seizure-free patients had an epileptic discharge in regular EEG recording [13–17]. The study showed that the LTM EEG recording is more sensitive to detect epileptiform activity than the regular EEG recording. Thus, we tend to get the point that LTM EEG has the advantage of monitoring in seizure-free individuals. It may help assess the recurrence risk after seizure [18].

EEG presentation is a commonly explored risk factor for relapse during AED treatment, especially following drug withdrawal. A previous report found that patients who did not undergo EEG follow-up examinations were usually related to a high recurrence rate following AED withdrawal [21]. Although these patients seem to be seizure-free, they might have experienced epileptiform activity. The LTM EEG recording may help find epileptiform activity and avoid seizure relapse.

The present study demonstrated that seizure-free patients with symptomatic epilepsy or abnormal imaging findings might have abnormal LTM EEG presentation. The study found 71 (27.1%) individuals having symptomatic epilepsy. Of these, patients with posttraumatic or postoperative epilepsy occupied a major position. Some studies found symptomatic etiology to be associated with an increased relative risk of relapse after AED withdrawal [7]. LTM EEG recording was helpful to detect the epileptiform activity and then adjust AED treatment for reducing the risk of relapse.

This study found that patients with posttraumatic epilepsy had 2.42 (95% CI 1.16–5.04) times abnormal EEG presentation compared with others. Abnormal EEG activity could predict seizure recurrence during AED therapy [22]. Even though the sample size in the present study was small, 10 (76.9%) patients with posttraumatic epilepsy were found to have abnormal EEG during long-term recording. The result agreed with other reports [23, 24] using continuous EEG recording and indicated that abnormal EEG disturbances were common. Proinflammatory cytokines, such as interleukin (IL)-1a and IL-1b, are implicated in the molecular cascade leading to neuronal injury after brain trauma. These patients are prone to have focal glial hyperplasia after 3 months or more [25–27]. Therefore, epileptiform discharges are induced, leading to drug-resistant epilepsy [28]. Consequently, patients with posttraumatic epilepsy should continue their AEDs to decrease the risk of seizure relapse for a longer time.

The present study found that patients with postoperative epilepsy had 3.36 (95% CI 1.31–8.6) times abnormal EEG presentation compared with others. Epileptiform discharges often occur beside the surgical site due to cortical injury and gliosis. The long-term prognosis of patients with epilepsy after cortical injury was good. However, LTM EEG should be followed up to decrease the risk of seizure relapse.

This study also demonstrated that the following factors did not predict greater susceptibility to LTM EEG presentation: age at onset of epilepsy, family history of epilepsy, history of febrile convulsions, severity of epilepsy before initiating AED treatment, seizure frequency, number of AEDs taken, and seizure-free period.

The result suggested no significant difference in patients who took one kind of AEDs or more. Patients who took two or more than two kinds of AEDs usually had refractory epilepsy previously. One study reported that the risk for relapse after a 24-month period of seizure remission was 46.7% at 3 years in a drug-resistant epileptic population [7]. A number of patients had abnormal EEG presentation. Despite seizure remission for a long time, they had a higher recurrence rate even if they still continued previous therapy. Thus, LTM EEG monitoring is necessary for these patients who took two or more AEDs. Although the patients were seizure-free for more than 2 years and had normal EEG presentation, they were advised to continue their AEDs for a longer time. A prospective study is ongoing aiming to provide more clinical hints.

This study had several limitations. First, it was a cross-sectional study. Therefore, prospective studies are needed to identify prognostic risk factors. Second, the patient population was small, making it difficult to assess less common potential risk factors, such as symptomatic etiology and AED type. Third, current LTM EEG recording is still not satisfactory. A more convenient and accurate EEG recording, such as video ambulatory EEG [29–31], should be applied in future prospective studies.

## Conclusions

LTM EEG was valuable of recording epileptiform discharges. The present study indicated that seizure-free patients with symptomatic epilepsy or abnormal imaging findings might have higher risk of abnormal LTM EEG presentation than those without symptomatic epilepsy or abnormal imaging findings. Of these, individuals with posttraumatic or postoperative epilepsy occupy a major position. These individuals need reevaluate the therapeutic strateagies and increase the hope to reach real seizure-free. The aim of following up LTM EEG is to detect the epileptiform activity and then adjust AED treatment for reducing the risk of relapse in seizure-free individuals.

## Data Availability

Characteristics of study population are included in the Tables and figure. Further data set could be obtained on request if required. Our data are deposited in our epilepsy center database.

## Abbreviations

AED: Antiepileptic drug
AEEG: Ambulatory EEG
ANOVA: One-way analysis of variance
CI: Confidence interval
EEG: Electroencephalogram
HR: Hazard ratio
VEEG: Video LTM EEG
